# Re-Programing Glucose Catabolism in the Microalga *Chlorella sorokiniana* under Light Condition

**DOI:** 10.3390/biom12070939

**Published:** 2022-07-04

**Authors:** Tingting Li, Na Pang, Lian He, Yuan Xu, Xinyu Fu, Yinjie Tang, Yair Shachar-Hill, Shulin Chen

**Affiliations:** 1 Department of Immunology, School of Basic Medical Sciences, Lanzhou University, Lanzhou 730000, China; litt@lzu.edu.cn; 2 Department of Biological Systems Engineering, Washington State University, Pullman, WA 99164, USA; pangna@msu.edu; 3 Department of Plant Biology, Michigan State University, East Lansing, MI 48823, USA; xuyuan5@msu.edu (Y.X.); yairhill@msu.edu (Y.S.-H.); 4 Department of Energy, Environmental and Chemical Engineering, Washington University, St. Louis, MO 63130, USA; lianheenvi@gmail.com (L.H.); yinjie.tang@seas.wustl.edu (Y.T.); 5 Department of Energy-Plant Research Laboratory, Michigan State University, East Lansing, MI 48823, USA; fuxinyu2@msu.edu

**Keywords:** microalgae, *Chlorella sorokiniana*, ^13^C-MFA, mixotrophic, heterotrophic, pool size

## Abstract

The microalga *Chlorella sorokiniana* has attracted much attention for lipid production and wastewater treatment. It can perform photosynthesis and organic carbon utilization concurrently. To understand its phototrophic metabolism, a biomass compositional analysis, a ^13^C metabolic flux analysis, and metabolite pool size analyses were performed. Under dark condition, the oxidative pentose phosphate pathway (OPP) was the major route for glucose catabolism (88% carbon flux) and a cyclic OPP–glycolytic route for glucose catabolism was formed. Under light condition, fluxes in the glucose catabolism, tricarboxylic acid (TCA) cycle, and anaplerotic reaction (CO_2_ fixation via phosphoenolpyruvate carboxylase) were all suppressed. Meanwhile, the RuBisCO reaction became active and the ratio of its carbon fixation to glucose carbon utilization was determined as 7:100. Moreover, light condition significantly reduced the pool sizes of sugar phosphate metabolites (such as E4P, F6P, and S7P) and promoted biomass synthesis (which reached 0.155 h^−1^). In addition, light condition increased glucose consumption rates, leading to higher ATP and NADPH production and a higher protein content (43% vs. 30%) in the biomass during the exponential growth phase.

## 1. Introduction

Nowadays, considering the increasingly depleted petrochemical resources and climate changes that are caused by the utilization of fossil fuels, scientists have started exploring the potential of algae as renewable energy alternatives. The green algae *Chlorella* spp. are a large group of eukaryotic, unicellular, and photosynthetic microorganisms, which have been widely studied as promising candidates for lipid-based biofuel feedstock [1,2,3]. Generally, microalgae can grow photoautotrophically by converting light and CO_2_ into biomass. Some species can also grow heterotrophically by assimilating organic carbon substrate as their only source of carbon and energy. One of the most attractive aspects of heterotrophic growth is the elimination of the requirement for light, which demonstrates the potential of high cell density fermentation [4,5]. Of particular interest is the mixotrophic mode of cultivation, in which organic carbon substrate and light are supplied simultaneously during the aerobic culture of microalgae. Mixotrophically cultured algal cells are capable of driving photosynthesis and heterotrophic metabolism to utilize both inorganic (CO_2_) and organic carbon substrates, thus leading to the synergistic effects of the two processes [6,7,8]. At present, mixotrophic cultivation has garnered a great deal of attention in various areas of research and applications, such as microalgae-derived biofuel feedstock and other bioproducts [9], dealing with eutrophic water [10], and wastewater treatment [11,12].

*C. sorokiniana* is one of the most important algal species that demonstrate high growth rates, tolerance against high-ammonia wastewater, and the capability to utilize diverse organic substrates [13]. Therefore, this species is promising for both wastewater treatment and bioproduction. In our previous study, *C. sorokiniana* displayed a higher specific growth rate in mixotrophic cultures compared to photoautotrophic and heterotrophic cultures [14]. The significant increase in growth rate in mixotrophic cultures (with light) relative to heterotrophic cultures (without light) is attributed to the re-utilization of the CO_2_ that evolved from the metabolic processes of organic substrates, such as the tricarboxylic acid (TCA) cycle and the oxidative pentose phosphate (OPP) pathway [14]. Additionally, the recycling of CO_2_ in mixotrophic cultures is commensurate with the adjusted photosynthetic capacity of mixotrophic cells. Mixotrophic *C. sorokiniana* cells in the exponential growth phase have enhanced photosynthetic activity, with a much higher oxygen evolution rate than that of photoautotrophic cells [15]. Moreover, non-photochemical quenching, which is a collection of processes that are thought to dissipate energy to protect the chloroplast from light damage, was not detected in *C. sorokiniana* cells that were cultured mixotrophically and was significantly reduced relative to cells in photoautotrophy [15].

However, further studies are needed to provide a molecular-level understanding of how photosynthesis could interfere with organic carbon assimilation, both of which co-exist in mixotrophic cells. In mixotrophic cells, CO_2_ that is harvested by photosynthesis and organic carbon substrates (i.e., glucose) that are assimilated through the heterotrophic process are all channeled into algal cells in parallel, so the question here is how the central pathways are partitioned and adjusted for sugar catabolism under light condition. The metabolic information reveals diverse metabolic routes that lead to improved biosynthesis.

## 2. Results

### 2.1. The Heterotrophic and Mixotrophic Growth of C. sorokiniana and its Metabolic Pseudo-Steady State

**Figure 1:**
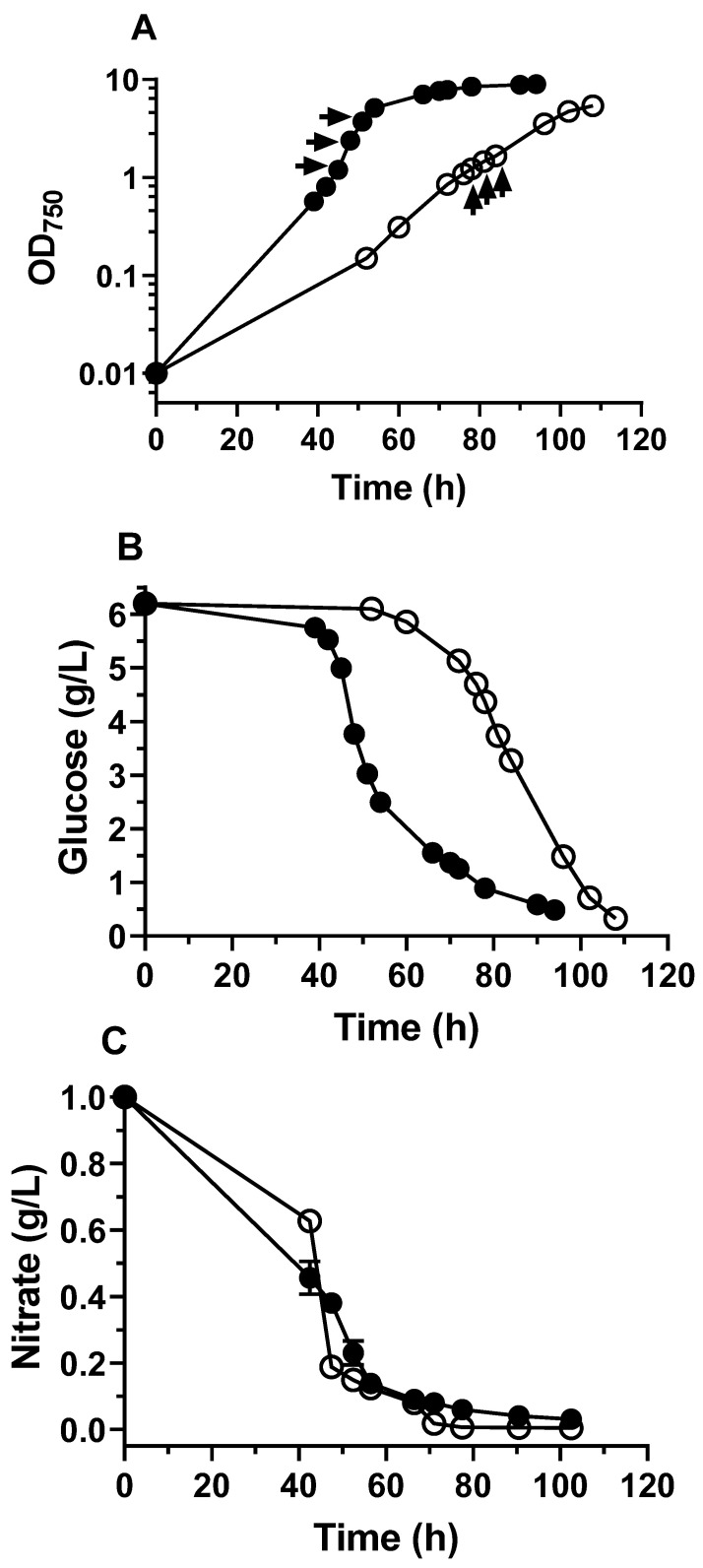
The growth of *C. sorokiniana* in batch cultures supplied with glucose under aerobic conditions. The cultures were grown mixotrophically (●) and heterotrophically (○). The arrows indicate the time points of sampling for the isotopic labeling patterns of proteinogenic amino acids. (**A**) The growth curves of *C. sorokiniana* under mixotrophic and heterotrophic conditions; (**B**) Glucose levels in mixotrophic and heterotrophic cultures; (**C**) Nitrogen consumption()n in mixotrophic and heterotrophic cultures. The error bars represent the standard deviations for the biological triplicates.

Cells grew much faster in the mixotrophic cultures (Figure 1A) with a specific growth rate of 0.155/h, which was two times that of the cells in the heterotrophic cultures (0.076/h). As the supplied organic substrate, glucose was consumed much faster in the mixotrophic cultures than in the heterotrophic cultures (Figure 1B), with glucose uptake rates of 1.82 and 1.39 mmol/g/h, respectively. Compared to the heterotrophic cultures, more nitrate was taken up in the mixotrophic cultures after 43 h of cultivation (Figure 1C), with 54.4% of nitrate being consumed. After 57 h, the remaining nitrogen contents in the two different cultures showed no significant differences.

To determine the pseudo-steady state metabolic period for the ^13^C-metabolic flux analysis (^13^C-MFA), the cultures were sampled at different time points to analyze the amino acid labeling of the cell biomass (Figure 1A). The labeling patterns in the biomass protein were relatively stable (standard deviations were around 0.01) among the samples that were taken during the exponential growth phase (Supplementary Materials, File S1). Therefore, the ^13^C-MFA in this study was implemented on ^13^C-labeled biomass samples that were taken during the early exponential growth phase. To access enough biomass to investigate biomass characterization and metabolite concentrations at the pseudo-steady state of *C. sorokiniana*, algal samples that were taken from the mid- and late exponential growth phases were analyzed.

### 2.2. Biomass Composition of C. sorokiniana

**Figure 2:**
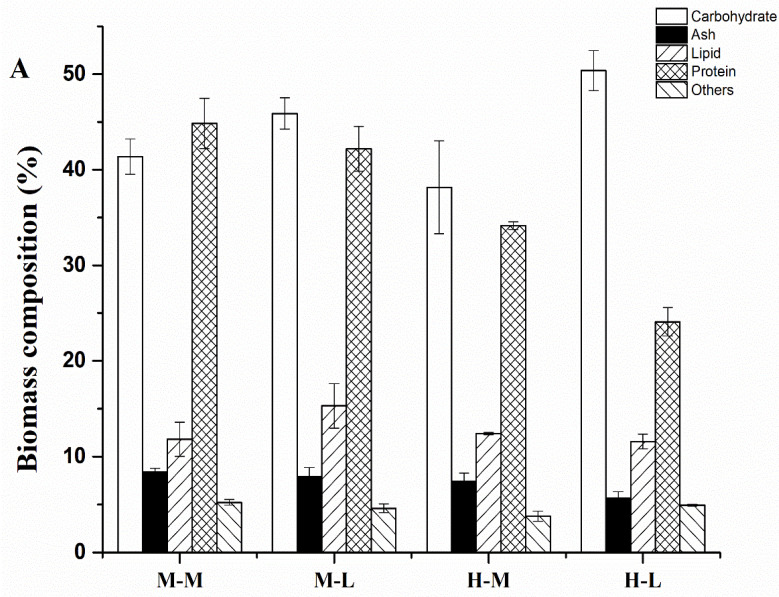

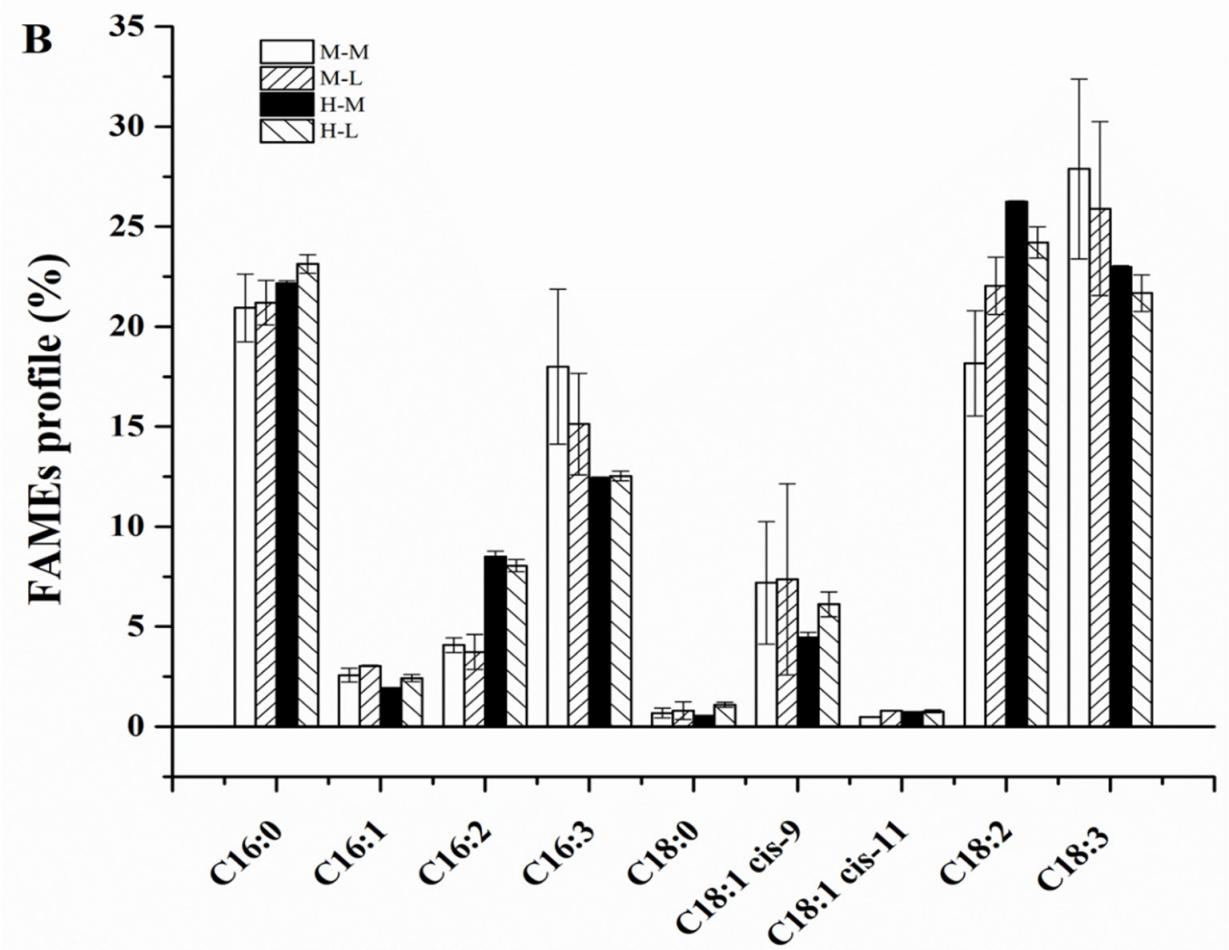
The biomass composition and FAMEs profile of *C. sorokiniana* when cultured mixotrophically and heterotrophically. (**A**) Biomass composition; (**B**) FAMEs profile. M-M, mid-exponential phase in mixotrophy; M-L, late exponential phase in mixotrophy; H-M, mid-exponential phase in heterotrophy; H-L, late exponential phase in heterotrophy.

Considering the potential of microalgae for bioenergy and high-value bioproduction, the macromolecular composition of algal biomass is an important factor to assess. Accordingly, the major components of the *C. sorokiniana* biomass at the pseudo-steady state were quantified, including carbohydrates, lipid, protein, ash, and “other” (Figure 2A). The results showed that the ash (<8.3%) and other (<5.2%) components made up just a small share of the overall biomass and that they showed no significant differences between *C. sorokiniana* cells that were cultured heterotrophically and those that were cultured mixotrophically. The major components in *C. sorokiniana* cells at the exponential growth phase were protein, carbohydrates, and lipid, which accounted for close to 90% of the overall biomass. Protein made up a large proportion of the algal biomass: 42–45% in mixotrophic cells and 24–34% in heterotrophic cells. This high content of protein guaranteed the utilization of protein as a great tracking carrier of isotopes for the metabolic flux analysis. Notably, the protein content in mixotrophic cells was relatively higher than that in heterotrophic cells and showed no apparent differences between the mid-exponential phase and the late exponential phase. In contrast, the protein content decreased significantly in heterotrophically cultured *C. sorokiniana* at the late exponential phase relative to the mid-exponential phase. Correspondingly, the carbohydrate content increased in heterotrophically cultured *C. sorokiniana* at the late exponential phase.

An analysis of the FAMEs profiles indicated that the major fatty acids in exponential-phase *C. sorokiniana* were C16 and C18. Among them, palmitic acid (C16:0), hexadecatrienoic acid (C16:3), linoleic acid (C18:2), and linolenic acid (C18:3) were the major fatty acids in *C. sorokiniana* (Figure 2B). The saturated fatty acid content (C16:0 and C18:0) in the algal lipid was 21.8–23.5% and showed no significant differences between the mid-exponential phase and the late exponential phase for algae that were cultured under the two conditions. The fatty acid content with three double bonds (C16:3 and C18:3) in mixotrophic algae was higher than that in heterotrophic algae, while the unsaturated fatty acid content with two double bonds (C16:2 and C18:2) was more accumulated in heterotrophic algae.

### 2.3. Metabolic Flux Analysis

**Figure 3:**
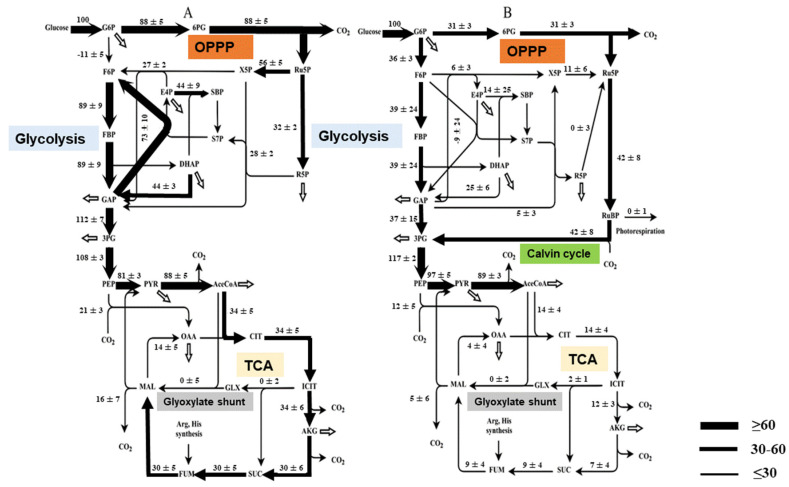
Flux distribution in the central metabolism of *C. sorokiniana*. (**A**) Heterotrophically cultured *C. sorokiniana* cells; (**B**) mixotrophically cultured *C. sorokiniana* cells. All estimated relative flux rates are shown next to the pathways, which are normalized to the glucose uptake rate. The standard deviation of each flux is shown, which was calculated based on the 95% confidence intervals (Supplementary Materials, File S3). The solid arrows represent the directions of the net fluxes and the wider arrows show higher flux rates. The open arrows represent the fluxes to the biomass.

Carbon and energy can only be obtained from exogenous glucose for heterotrophic cells, yet they can be derived from both exogenous glucose and photosynthesis for mixotrophic cells [14]. To thoroughly understand the carbon distribution and metabolite flux in *C. sorokiniana* cells that were cultured mixotrophically relative to those that were cultured heterotrophically, we generated quantitative flux maps of *C. sorokiniana* cells that were cultured under these two conditions by performing a ^13^C-MFA (Figure 3). The relevant reactions are listed in the Supplementary Materials, File S2. The net fluxes were normalized to a glucose uptake rate of 100 in the mixotrophic and heterotrophic cells.

In the heterotrophic cells, 88% of the carbon flux entered the oxidative pentose phosphate (OPP) pathway by oxidizing glucose 6-phosphate (G6P) into 6-phosphogluconate (6PG), followed by decarboxylation into ribulose 5-phosphate (Ru5P). Ru5P involved in a non-oxidative pentose phosphate (PP) pathway, yielded intermediates in very high flux; in particular, carbon flux (73%) from glyceraldehyde 3-phosphate (GAP) and sedoheptulose 7-phosphate (S7P) into erythrose 4-phosphate (E4P) and fructose 6-phosphate (F6P). Noticeably, a significant flux (11%) of F6P was channeled into G6P by the reverse reaction of glucose 6-phosphate isomerase (PGI), which formed a glucose degradation loop. Furthermore, two important steps in the glycolysis pathway (the phosphorylation of F6P into fructose 1,6-bisphosphate (FBP) and the lysis of FBP into dihydroxyacetone phosphate (DHAP) and glyceraldehyde 3-phosphate (GAP)) displayed an extremely high carbon flux of close to 90% in the heterotrophic cells.

In contrast, the mixotrophic cells exhibited a distinct carbon flux pattern during central metabolism. The OPP pathway, non-oxidative PP pathway, and glycolysis pathway were much less active in the mixotrophic cells, with carbon fluxes that were significantly lower than those in the heterotrophic cells. However, it was observed that the flux of CO_2_ fixation throughout the Calvin–Benson cycle was 42% of the glucose input flux in the mixotrophic *C. sorokiniana* cells. In other words, the ratio of carbon fixation to glucose carbon utilization was determined as 7:100.

The carbon fluxes from glycolysis and the PP pathways, including the Calvin–Benson cycle in mixotrophic cells, are eventually channeled into the tricarboxylic acid (TCA) cycle. The TCA cycle begins with a reaction that combines the two-carbon acetyl-CoA with the four-carbon oxaloacetate (OAA) to generate the six-carbon citrate (CIT), which is a primary source of energy and reducing equivalents in aerobic organisms. As shown in Figure 3, the proportion of acetyl-CoA that was channeled into the TCA cycle was 38% in the heterotrophic cells and 15% in the mixotrophic cells. Additionally, the anaplerotic pathway from malate to pyruvate through the malic enzyme was less active in the mixotrophic cells, leading to less CO_2_ dissipation.

### 2.4. Relative Pool Sizes of Metabolites in C. sorokiniana

The intracellular metabolite concentrations in organisms exert influences on biochemical reactions, free energy, and metabolite flux organization [16]. This study further investigated the responses of *C. sorokiniana* cells under different culture conditions at the metabolite level. Taking fully ^13^C- labeled *E. coli* extracts as the internal standard, the pool sizes of *C. sorokiniana* were measured using the isotopic ratio method and the relative ratio of the metabolite pool sizes was normalized by the amount of biomass.

**Figure 4:**
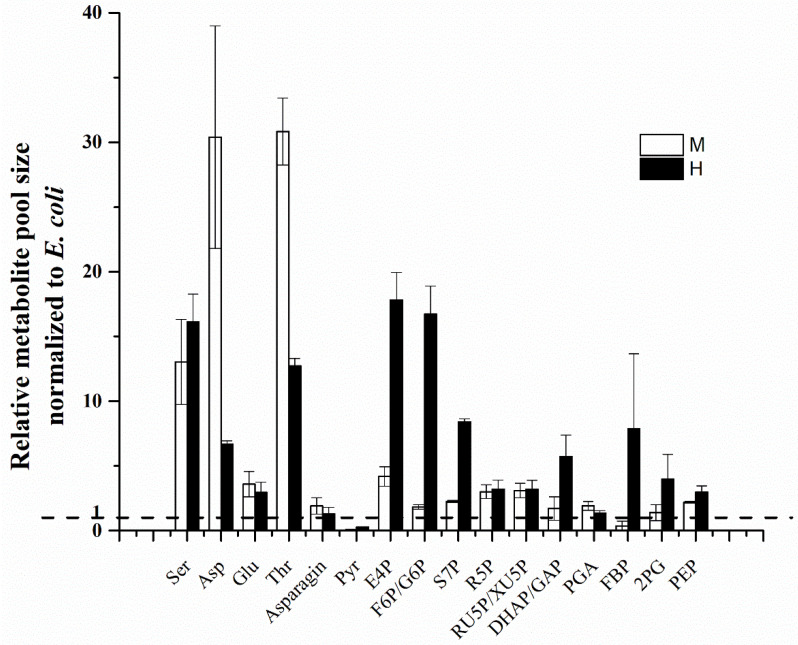
The relative pool size of *C. sorokiniana* normalized to *E. coli* K-12 under mixotrophic (M) and heterotrophic (H) conditions. A ratio of 1 indicates the same metabolite concentrations (normalized to gram biomass) in *C. sorokiniana* and *E. coli*.

Compared to *E. coli*, most metabolites were more abundant in *C. sorokiniana* cells, except for pyruvate and mixotrophy-derived FBP (Figure 4). The concentrations of E4P, F6P/G6P, S7P, and DHAP/GAP were 4.3-, 9.1-, 3.7- and 3.4-fold higher in the heterotrophic cells than the mixotrophic cells, respectively. Additionally, the mixotrophic cells had significantly larger pool sizes of aspartate (Asp) and threonine (Thr) than the heterotrophic cells, in which were 4.5- and 2.4-fold higher, respectively. This result indicated that light condition could more effectively drive sugar phosphate metabolites toward building block synthesis in the biomass.

## 3. Discussion

### 3.1. The Phenotypes of Heterotrophically and Mixotrophically Cultured C. sorokiniana

The growth behavior of *C. sorokiniana* was quite distinct under mixotrophic conditions relative to heterotrophic conditions. In the mixotrophic cultures, the specific growth rate was significantly higher and glucose was consumed much faster. Compared to the heterotrophic cells, the carbohydrate content in the mixotrophic cells was not significantly different at the mid- and late exponential growth phases. Furthermore, protein was dominant in the mixotrophic cells at the exponential growth phase, accounting for 42–45% of the biomass composition. The higher protein content in the mixotrophic cells serviced the fast growth of the algal cells at the exponential phase. Contrarily, the protein content in the exponential-phase heterotrophic cells was much lower (particularly in cells at the late exponential phase), with a value of 24%, indicating probable nutrient limitation and/or the accumulation of adverse factors for cell growth starting at the late exponential growth phase. The differences between the protein contents of the cells in the mixotrophic and heterotrophic cultures were significant. The above results highly corroborated the better growth performance of algal cells that were cultured mixotrophically than those that were cultured heterotrophically, thereby demonstrating the advantages of mixotrophic cultivation as an efficient strategy for algal bioproduct synthesis.

**Figure 5:**
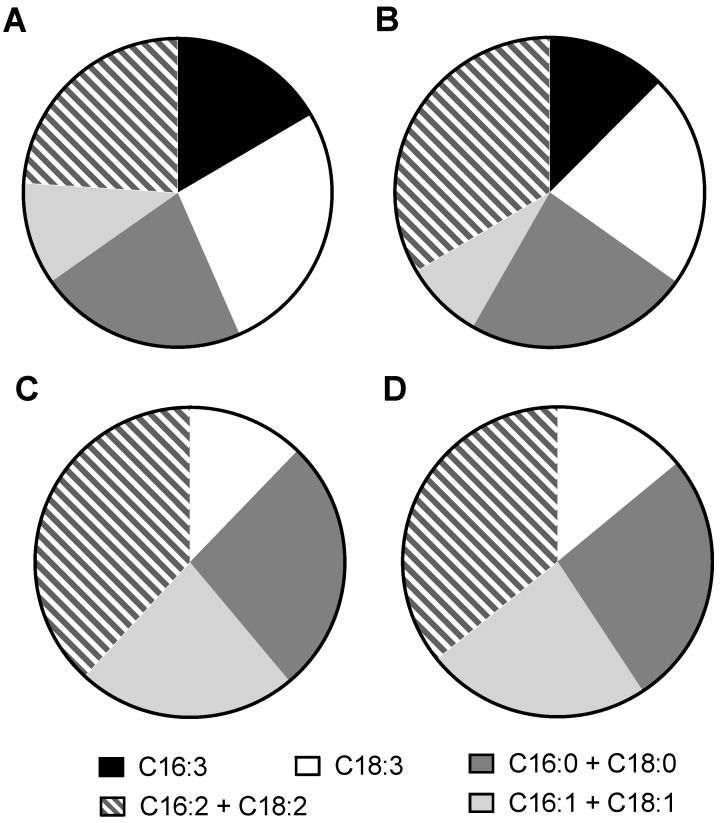
Changes in the fatty acids of *C. sorokiniana* at different growth phases under mixotrophic and heterotrophic conditions. Fatty acids were analyzed from this and a previous study: (**A**) mixotrophic cells at the exponential phase (this study); (**B**) heterotrophic cells at the exponential phase (this study); (**C**) mixotrophic cells at the stationary phase (Li et al., 2014); (**D**) heterotrophic cells at the stationary phase (Li et al., 2014).

Fatty acid production was dominated by C16 and C18 in the exponential-phase *C. sorokiniana* cells that were cultured heterotrophically and mixotrophically. As with those at the exponential phase, *C. sorokiniana* cells that were cultured heterotrophically and mixotrophically at the stationary phase also possessed fatty acids that mainly had carbon chain lengths of 16 and 18 [14]. The percentages of saturated fatty acids (C16:0 and C16:2) in the total fatty acids at the stationary phase were similar to those at the exponential phase, no matter whether *C. sorokiniana* was cultured heterotrophically or mixotrophically (Figure 5). However, the unsaturated fatty acid profiles of *C. sorokiniana* at the exponential phase and stationary phase were distinctly divergent. The barely detectable C16:3 in *C. sorokiniana* at the stationary phase (relative to the 16.6–12.5% in the total fatty acid content at the exponential phase) was particularly noteworthy (Figure 5) and clearly reflected the significant role of C16:3 during active cell growth. Additionally, the proportion of C18:3 in *C. sorokiniana* at the exponential phase was much higher than that at the stationary phase. Conversely, the proportion of C16:1 and C18:1 was significantly pronounced in *C. sorokiniana* cells that were harvested at the stationary phase relative to those that were harvested at the exponential phase. The fatty acid distributions in *C. sorokiniana* at different growth phases provided us with a better understanding of lipid metabolism in microalgae and its potential for applications in bioenergy and other areas.

### 3.2. Carbon Metabolic Fluxes

The mixotrophic and heterotrophic *C. sorokiniana* cells exhibited distinct carbon flux patterns throughout central metabolism. In the flux distribution of the heterotrophic cells, 88% was channeled into the OPP pathway (Figure 3). The high flux through the OPP pathway yielded a large amount of NADPH, as well as excess pentoses (i.e., Ru5P and Ru5P). The pentoses were greatly catabolized via the non-oxidative PP pathway, as demonstrated by the high flux together with the large sizes of the metabolites that were involved in the non-oxidative PP pathway, such as E4P, S7P, etc. (Figure 4). Interestingly, the heterotrophic *C. sorokiniana* cells contained an active cycle via the PP pathway, within which G6P was extensively degraded by the OPP pathway for NADPH production and was replenished from F6P that was catalyzed by the PGI. This carbohydrate degradation cycle was not discovered in other heterotrophically cultured eukaryote microalgae, such as *Chlorella protothecoides* [17] or *Crypthecodinium cohnii* [18].

Considering that the OPP pathway with its overwhelming carbon flux was the principal source of CO_2_ production in the heterotrophic cells, which was strongly negatively correlated with biomass synthesis, the significantly lower flux (31%) of the OPP pathway in the mixotrophic cells converted glucose into algal biomass more efficiently. Reductions in the unusually large fluxes in the OPP pathway under high light condition has also been observed when developing *Camelina* seeds, thereby raising the conversion efficiency of substrates [19].

In the mixotrophic cells, the Calvin–Benson cycle became active and the flux of CO_2_ fixation throughout the Calvin–Benson cycle was much higher than that in the OPP pathway. The active Calvin–Benson cycle was consistent, with attainable photosynthetic capability and active carboxylase activity for RuBisCO in the mixotrophic *C. sorokiniana* cells, as observed in our previous study [15]. The CO_2_ production from the OPP pathway is one of the principal sources of carbon loss. Nevertheless, CO_2_ fixation via RuBisCO could completely compensate for this CO_2_ dissipation, resulting in a reasonable explanation for the fact that the mixotrophic cells could assimilate glucose more efficiently and grow faster. Decreased OPP activity and active RuBisCO have also been observed in green seeds under light condition and active RuBisCO has been proven to mostly recapture CO_2_ internally rather than fix external CO_2_ [20]. Contrarily, because of the inactive Calvin–Benson cycle together with the higher fluxes in the OPP pathway, a more exogenous glucose carbon loss was presented in the form of CO_2_ release in the heterotrophic cells, leading to their lower efficiency in converting glucose into cell biomass. Although an alternative CO_2_ assimilation pathway through PEP carboxylase exists in both mixotrophic and heterotrophic cells, it has relatively lower fluxes in both cell types.

As the primary source of energy and reducing equivalents in aerobic organisms, the TCA cycle was much less active in the mixotrophic cells than the heterotrophic cells (Figure 3). Consistent with our findings, a genome-scale metabolic model for *Chlorella vulgaris* predicted that most enzymes in the TCA cycle would be highly active during heterotrophic growth and a few of them would not be active during photoautotrophic or mixotrophic growth. For example, the mitochondrial isocitrate dehydrogenase is only active during heterotrophic growth [21]. The low fraction of acetyl-CoA that was distributed into the TCA cycle laid the foundation for the down-regulation of the TCA cycle in the mixotrophic cells. Considering its contribution to energy generation and metabolite provision for cell metabolism and its significantly lower fluxes, as well as the functional photosynthetic activity, the TCA cycle in the mixotrophic cells principally committed to metabolite biosynthesis instead of energy production, which was similar to photoautotrophic microbes [22,23].

In our study, the glyoxylate shunt activity in *C. sorokiniana* was not found in either of the culture conditions. The glyoxylate shunt bypasses the metabolic steps in the TCA cycle from isocitrate to malate. It serves an anaplerotic function for the cell growth on acetate or fatty acids and replenishes the carbon skeletons that are withdrawn from the TCA cycle for biosynthesis. This pathway was found to be active when the green alga *Chlamydomonas reinhardtii* was grown on acetate [24] but was inactive in heterotrophic *Chlorella protothecoides* [17]. Additionally, it also showed activity in both the heterotrophic and mixotrophic metabolism of the cyanobacterium *Synechocystis* species PCC6803 [25]. Due to the absence of the glyoxylate shunt in *C. sorokiniana*, the flux through PEP carboxylase exclusively replenished the metabolic pools of the TCA cycle, with 75% of the OAA being channeled from this flux into the mixotrophic cells.

### 3.3. ATP and NADPH Metabolism

Based on the quantitative results of the ^13^C-MFA, the production and consumption of ATP and NADPH could be analyzed in *C. sorokiniana* cells that were cultured heterotrophically and mixotrophically. The ATP generation from the central metabolism of glycolysis and the TCA cycle was far less than the ATP demand of the heterotrophic and mixotrophic cells (Table 1). Nevertheless, the principal ATP could be derived from oxidation phosphorylation through the respiratory chain by converting NADH into ATP. Assuming a P/O ratio of 2 (the moles of ATP formed per oxygen atom: NADH → 2ATP), the ATP formation fluxes via oxidation phosphorylation were 596 mol mol glucose^−1^ h^−1^ in the heterotrophic cells and 356 mol mol glucose^−1^ h^−1^ in the mixotrophic cells. The ATP that was collected from the oxidation phosphorylation was more than enough to compensate for the shortage of ATP in the heterotrophic cells, whereas it was inadequate to make up the ATP deficit to meet the ATP demand in the mixotrophic cells. Considering that the photosynthetic electron transport chain also generates a proton motive force, which drives the production of ATP from ADP and Pi by the ATP synthase [26], photosynthesis in the mixotrophic *C. sorokiniana* cells was another non-negligible route for ATP generation.

As shown in Table 1, the NADPH in the heterotrophic *C. sorokiniana* cells was provided by the OPP and anaplerotic pathways. The OPP pathway, with NADPH as a byproduct from reactions that were catalyzed by G6P dehydrogenase and 6PG dehydrogenase, is a primary NADPH synthesis route, which supplied up to 92% of the NADPH in the heterotrophic *C. sorokiniana* cells (Table 1). Contrarily, the OPP pathway could only fulfill 21% of the NADPH requirement in the mixotrophic cells. As in the heterotrophic cells, NADPH could also be obtained from the anaplerotic pathway, which is the conversion process of malate into pyruvate when catalyzed by the malic enzyme, but the amount was subtle, making up less than 2% of the overall NADPH demand. Consequently, the need for NADPH far exceeded the amount of NADPH that was produced by the central metabolism in the mixotrophic cells and there had to be a large supply of NADPH to fulfill the biosynthetic demands. The photosynthetic activity in the mixotrophic *Chlorella* cells was proven to be intensely active [15] and, as a primary photosynthetic reductant, NADPH could be generated in abundance from a linear photosynthetic electron transport chain. Hence, photosynthetic electron transport accounted for a major fraction of the NADPH production in the mixotrophic *C. sorokiniana* cells.

## 4. Conclusions

The present study monitored the physiological variations in *C. sorokiniana* cells that were cultured mixotrophically and heterotrophically using a biomass component analysis, a ^13^C metabolic fluxes analysis, and the quantification and pool sizes of metabolites. Heterotrophic *C. sorokiniana* generated more ATP in the TCA cycle and more NADPH in the OPP pathway. By comparison, the carbon fluxes in glycolysis and the PP pathway were greatly limited in mixotrophic cells. Together with a less active TCA cycle, mixotrophic cells exhibited less carbon loss in the form of CO_2_ release and converted exogenous carbon substrate efficiently. Moreover, significant CO_2_ fixation via RuBisCO was observed and highly active photosynthesis played a key role in the supply of ATP and NADPH in mixotrophic cells. Our study provides a comprehensive understanding of carbon and energy fluxes in mixotrophically and heterotrophically cultured *C. sorokiniana*. 

## 5. Methods

### 5.1. Microalgal Cultivation and Labeling Experiments 

The green microalga *C. sorokiniana* (UTEX 1602) was obtained from the UTEX Culture Collection of Algae at the University of Texas at Austin (USA). A modified Kuhl medium was used, as described previously in [14]. The algal cells were routinely cultured at 25 ^o^C in 250-mL flasks containing 150 mL of the modified Kuhl medium, which was agitated with filter-sterilized air enriched with CO_2_ to 1%. Illumination was provided by cool white fluorescent lamps to enable a constant light intensity of 100 µmol m^−2^ s^−1^. For the labeling experiments, 6 g/L D-[1,2-^13^C_2_] glucose with a purity of 99% (Sigma-Aldrich, St. Louis, MO, USA) was used. Inocula from an unlabeled *C. sorokiniana* photoautotrophic culture were added into 50 mL of medium, which contained ^13^C-labeled glucose in 250-mL Erlenmeyer flasks. All cultures were started with an initial OD_750_ value of 0.01 to minimize the inoculation effects and were then grown at 25 °C under constant illumination (100 µmol m^−2^ s^−1^) on an orbital shaker at 150 rpm. For each experimental condition, three replicates were performed and the standard deviation was calculated.

### 5.2. Carbon and Nitrogen Consumption Analysis

The use of carbon and nitrogen sources was tracked in the algal cultures. The algal suspensions under different cultivation conditions were centrifuged to remove cells and were then filtered with 0.2-μM pore size filter discs. The filtered media were used to analyze the remaining glucose and KNO_3_. The glucose in media was analyzed by HPLC (Varian Prostar) using a refractive index detector (Model 350, Varian), as described by Pang et al. [27], and the nitrogen consumption was determined using the H_2_SO_4_–salicylic acid method [28].

### 5.3. Biomass Composition Characterization 

The biomass composition of *C. sorokiniana* that was cultured under different conditions at the mid- and late exponential phases, including carbohydrates, lipid, protein, ash, and “other”, was characterized using an unlabeled algal biomass. The carbohydrate content in the algal biomass was determined using a modified method of two-stage sulfuric acid hydrolysis [29,30]. The soluble carbohydrates were analyzed by an HPLC (Agilent 1100) after the hydrolysis, which was equipped with a Bio-rad Aminex HPX-87H analytical column and a refractive index detector [31]. The protein content was determined using elemental analysis and a nitrogen-to-protein (NTP) conversion factor of 4.87 [32]. The elemental analysis was performed using a TruSpec micro-elemental analyzer (CHN628 Series, LECO Corporation) with the corresponding ASTM procedure (D-5291, E-777, and E-778). The relative compositions of carbon, nitrogen, and hydrogen were obtained as a percentage of the mass. The protein content was calculated by multiplying the nitrogen content by the standard NTP. The ash and other components in the dry algal biomass were measured by thermogravimetric analysis using a TGA/SDTA 851e thermogravimetric analyzer (Mettler Toledo, Switzerland), which was equipped with a STARe data analysis system [33]. The lipid content was determined as previously described in [34].

### 5.4. Relative Pool Sizes of Metabolites 

The relative pool sizes of the metabolites were measured in *C. sorokiniana* cells that were cultured under different conditions at the exponential phase and were then analyzed using an MS isotopomer ratio method for semiquantitative metabolomics, with fully labeled *E. coli* extracts as the internal standards [35]. The relative metabolite pool sizes were estimated by mixing a known amount of fully ^13^C-labeled *E. coli* biomass with unknown and unlabeled *Chlorella* cultures. The isotopic ratio of each labeled and unlabeled metabolite was normalized by the amount of *E. coli* and algal biomass, respectively.

### 5.5. Metabolite Quenching, Extraction, and Analysis

After the ^13^C-pulse experiments, the samples in the centrifuge tubes were immediately quenched in liquid nitrogen and rapidly swirled, followed by thawing at 4 °C. The samples were then centrifuged at 6000 × *g* for 10 min at 4 °C, and biomass pellets were obtained for metabolite extraction and mass spectrometry analysis.

Metabolites in the biomass were extracted using a modified method [36]. Specifically, 300 µL of ice-cold CHCl_3_/CH_3_OH (3:7, *v*/*v*) was added to each sample with vigorous shaking and then the samples were incubated at −20 °C for 2 h with occasional mixing. Water-soluble metabolites were extracted by adding 350 µL of water with vigorous shaking and centrifugation at 4 °C and 16,000× *g* for 10 min. The upper methanol–water phase was recovered, lyophilized, and aliquoted for reverse-phase LC-MS/MS, anion exchange LC-MS/MS, and GC-MS [37]. Most of the Calvin cycle intermediates were analyzed using reverse-phase LC-MS/MS with an ACQUITY UPLC pump system (Waters, Milford, MA, USA) coupled with a Quattro Premier LC-MS/MS system (Waters, Milford, MA, USA). Mass spectra were acquired using multiple reaction monitoring (MRM) in negative electrospray ionization (ESI) mode. Anion exchange LC-MS/MS was used to analyze phosphorylated metabolites with an ACQUITY UPLC pump system (Waters, Milford, MA, USA) coupled with a Xevo ACQUITY TQ Triple Quadrupole Detector (Waters, Milford, MA, USA). Mass spectra were also acquired using MRM in negative ESI mode. Amino acids and organic acids in the algae biomass were derivatized by methoximation, followed by tertbutyldimethylsilylation. The derivatized samples were analyzed by GC-MS with an Agilent 7890 GC system (Agilent, Santa Clara, CA, USA) coupled with an Agilent 5975C inert XL Mass Selective Detector (Agilent, Santa Clara, CA, USA).

### 5.6. GC-MS Analysis of ^13^C Proteinogenic Amino Acids

The labeling of the proteinogenic amino acids was measured using GC-MS, as previously described in [38]. Briefly, the biomass was harvested by centrifugation and was then washed twice with a 0.9% NaCl solution. The pellets were then hydrolyzed with 6 M HCl at 100 °C for 24 h. The resulting mixtures were subsequently air-dried and derivatized with *N-tert*-butyldimethylsilyl-*N*-methyltrifluoroacetamide (Sigma-Aldrich) at 70 °C for 1 h, then analyzed by GC-MS (Agilent, CA, USA) with a DB5 column. The GC thermal programs were described in a previous report [38]. A published algorithm was used to calculate the MS data for all derivatized metabolites [39]. Due to overlapping peaks or product degradation, several amino acids (proline, arginine, cysteine, and tryptophan) were not used for the flux analysis [40]. The MS data ([M-57], [M-159] or [M-85], and [f302]) of key amino acids are shown in the Supplementary Materials, File S1. The isotopomer labeling fractions that are designated as M0, M1, M2, etc. represent fragments containing amino acids that were unlabeled, singly labeled, doubly labeled, etc.

### 5.7. ^13^C Metabolic Flux Analysis

The ^13^C-MFA was carried out based on the labeling patterns of the proteinogenic amino acids in mixotrophically and heterotrophically cultured *C. sorokiniana* that was supplied with [1,2-^13^C_2_] glucose. The major metabolite reactions are shown in the Supplementary Materials, File S2. The labeling distributions of key amino acids were relatively stable among the samples that were taken during the exponential growth phase (Supplementary Materials, File S1), indicating a pseudo-steady state metabolic period.

By normalizing the glucose uptake rate to a value of 100 (unitless), the ^13^C-MFA profiled the relative metabolic fluxes throughout the central metabolism. The relative fluxes were solved by minimizing a quadratic error function that calculated the differences between the predicted and measured isotopomer patterns **(**Supplementary Materials, File S3) using the built-in “fmincon" MATLAB function. To calculate the 95% confidence intervals, we perturbed the data 500 times and recalculated the fluxes. The upper and lower bounds were determined by the top and bottom 2.5% values.

## Figures and Tables

**Table 1:** A comparison of the energy molecule production and consumption from different pathways.

	M	H	M	H	M	H
Pathway	ATP		NADH		NADPH	
Glycolysis	−4	4	37	112	n/a	n/a
TCA Cycle	7	30	113	167	n/a	n/a
OPP/Calvin Cycle	−42	0	n/a	n/a	61	175
Anaplerotic Pathway	n/a	n/a	n/a	n/a	5	16
Biomass	−382	−274	28	20	−284	−191
Overall Balance	−421	−240	178	298	−219	0

Note: all values are normalized to a glucose uptake rate of 100 mol h^−1^ in both mixotrophic cells (M) and heterotrophic cells (H).

## Data Availability

All data generated or analyzed during this study are included in this published article and its supplementary materials.

## Supplementary Materials

The following supporting information can be downloaded at: https://www.mdpi.com/article/10.3390/biom12070939/s1. File S1: Labeling patterns of proteinogenic amino acids in *C. sorokiniana*, File S2: Metabolic reactions in *C. sorokiniana*,File S3: Relative flux distributions in *C. sorokiniana*.

## Abbreviations

TCA, tricarboxylic acid; OPP, oxidative pentose phosphate; PP, pentose phosphate; ^13^C-MFA, ^13^C-metabolic flux analysis; 3PG, 3-phosphoglycerate; AKG, α-ketoglutarate; CIT, citrate; DHAP, dihydroxyacetone phosphate; E4P, erythrose 4-phosphate; F6P, fructose 6-phosphate; FUM, fumarate; G6P, glucose 6-phosphate; GAP, glyceraldehyde 3-phosphate; GLX, glyoxylate; HIS, histidine; ICIT, isocitrate; MAL, malate; OAA, oxaloacetate; PEP, phosphoenolpyruvate; PYR, pyruvate; R5P, ribose 5-phosphate; Ru5P, ribulose 5-phosphate; RuBP, ribulose 1,5-diphosphate; S7P, sedoheptulose 7-phosphate; SUC, succinate; X5P, xylulose 5-phosphate; FBP, fructose 1,6-bisphosphate; R5P, ribose 5-phosphate; AceCoA, acetyl coenzyme A; SucCoA, succinyl coenzyme A; SBP, sedoheptulose 1,7-bisphosphate; 6PG, 6-phosphogluconate.
