# Supplementary material for: Re-Programing Glucose Catabolism in the Microalga Chlorella sorokiniana under Light Condition
Source: Biomolecules. 2022 Jul 4;12(7):939. doi: 10.3390/biom12070939 (PMC9313030; doi:10.3390/biom12070939)
Supplement: Supplementary file 1 [file biomolecules-12-00939-s001.zip › Supplementary Materials, File S1.pdf]

Supplementary Materials, File S1. Labeling patterns of proteinogenic amino acids in *C. sorokiniana*

	Mixotrophic						Heterotrophic					
	M1-1	M1-2	M2-1	M2-2	M3-1	M3-2	H1-1	H1-2	H2-1	H2-2	H3-1	H3-2
'Alanine [M-57]'												
'M+0'	0.513	0.516	0.521	0.516	0.496	0.496	0.572	0.568	0.570	0.571	0.568	0.567
'M+1'	0.108	0.116	0.118	0.120	0.133	0.137	0.175	0.175	0.178	0.176	0.176	0.178
'M+2'	0.353	0.343	0.337	0.336	0.332	0.327	0.241	0.244	0.240	0.241	0.243	0.242
'M+3'	0.026	0.025	0.023	0.027	0.038	0.040	0.012	0.013	0.012	0.012	0.013	0.013
'Alanine [M-85]'												
'M+0'	0.541	0.548	0.554	0.551	0.542	0.540	0.626	0.623	0.623	0.623	0.623	0.621
'M+1'	0.119	0.128	0.132	0.132	0.132	0.136	0.205	0.205	0.210	0.208	0.206	0.208
'M+2'	0.340	0.324	0.313	0.316	0.326	0.323	0.169	0.172	0.167	0.169	0.171	0.172
'Glycine [M-57]'												
'M+0'	0.560	0.581	0.573	0.572	0.555	0.569	0.666	0.664	0.668	0.666	0.668	0.669
'M+1'	0.366	0.356	0.366	0.366	0.349	0.340	0.303	0.304	0.302	0.304	0.303	0.302
'M+2'	0.074	0.063	0.061	0.062	0.096	0.091	0.031	0.032	0.030	0.030	0.030	0.029
'Glycine [M-85]'												
'M+0'	0.628	0.654	0.652	0.654	0.636	0.650	0.805	0.803	0.806	0.805	0.809	0.810
'M+1'	0.372	0.346	0.348	0.346	0.364	0.350	0.195	0.197	0.194	0.195	0.191	0.190
'Valine [M-57]'												
'M+0'	0.287	0.289	0.293	0.291	0.287	0.290	0.380	0.379	0.380	0.379	0.380	0.380
'M+1'	0.118	0.127	0.132	0.135	0.133	0.136	0.231	0.229	0.232	0.230	0.233	0.232
'M+2'	0.376	0.369	0.365	0.363	0.365	0.360	0.271	0.272	0.270	0.271	0.269	0.270
'M+3'	0.087	0.090	0.089	0.092	0.092	0.093	0.077	0.078	0.078	0.078	0.078	0.078
'M+4'	0.124	0.116	0.113	0.111	0.114	0.112	0.040	0.040	0.039	0.041	0.038	0.038
'M+5'	0.008	0.008	0.007	0.008	0.009	0.009	0.001	0.002	0.002	0.002	0.002	0.001
'Valine [M-159]'												
'M+0'	0.301	0.306	0.309	0.309	0.307	0.308	0.408	0.408	0.407	0.407	0.410	0.408
'M+1'	0.126	0.137	0.143	0.146	0.142	0.146	0.261	0.258	0.262	0.259	0.264	0.263
'M+2'	0.370	0.362	0.358	0.354	0.358	0.356	0.233	0.235	0.233	0.234	0.230	0.232
'M+3'	0.084	0.087	0.086	0.088	0.086	0.087	0.071	0.071	0.071	0.072	0.070	0.070
'M+4'	0.118	0.108	0.104	0.103	0.106	0.103	0.027	0.028	0.027	0.027	0.026	0.027
'Leucine [M-85]'												
'M+0'	0.179	0.183	0.185	0.185	0.182	0.185	0.288	0.288	0.288	0.287	0.289	0.288
'M+1'	0.197	0.206	0.211	0.213	0.210	0.212	0.309	0.307	0.310	0.309	0.311	0.311
'M+2'	0.270	0.271	0.269	0.269	0.270	0.270	0.241	0.241	0.240	0.241	0.240	0.240
'M+3'	0.202	0.198	0.198	0.197	0.198	0.197	0.116	0.117	0.116	0.117	0.116	0.116
'M+4'	0.100	0.096	0.092	0.092	0.094	0.092	0.037	0.037	0.037	0.037	0.036	0.036
'M+5'	0.052	0.047	0.045	0.044	0.046	0.044	0.009	0.009	0.009	0.009	0.008	0.008
'Isoleucine [m-85]'												
'M+0'	0.237	0.227	0.227	0.226	0.228	0.225	0.298	0.305	0.295	0.301	0.302	0.296
'M+1'	0.164	0.177	0.185	0.189	0.186	0.189	0.304	0.303	0.303	0.304	0.306	0.304

'M+2'	0.325	0.317	0.314	0.310	0.314	0.310	0.248	0.246	0.250	0.247	0.246	0.249
'M+3'	0.141	0.148	0.149	0.152	0.148	0.153	0.108	0.106	0.109	0.107	0.106	0.109
'M+4'	0.109	0.105	0.101	0.098	0.100	0.099	0.036	0.035	0.036	0.035	0.034	0.036
'M+5'	0.024	0.025	0.025	0.025	0.024	0.024	0.006	0.006	0.006	0.006	0.006	0.006
'Serine [M-57]'												
'M+0'	0.482	0.477	0.493	0.478	0.449	0.438	0.574	0.544	0.570	0.580	0.580	0.569
'M+1'	0.171	0.177	0.180	0.190	0.216	0.222	0.215	0.223	0.217	0.213	0.216	0.217
'M+2'	0.318	0.317	0.303	0.304	0.291	0.292	0.203	0.222	0.205	0.200	0.197	0.204
'M+3'	0.030	0.030	0.024	0.028	0.044	0.049	0.007	0.011	0.008	0.007	0.007	0.009
'Serine [M-159]'												
'M+0'	0.529	0.523	0.545	0.531	0.506	0.492	0.649	0.616	0.644	0.659	0.656	0.645
'M+1'	0.190	0.197	0.195	0.205	0.247	0.258	0.226	0.243	0.229	0.219	0.225	0.231
'M+2'	0.281	0.280	0.261	0.264	0.247	0.250	0.125	0.141	0.127	0.122	0.119	0.124
'Phenylalanine [M-57]'												
'M+0'	0.201	0.194	0.179	0.198	0.174	0.197	0.283	0.257	0.278	0.288	0.286	0.279
'M+1'	0.116	0.123	0.121	0.129	0.120	0.130	0.225	0.217	0.226	0.227	0.229	0.226
'M+2'	0.314	0.308	0.302	0.306	0.302	0.303	0.269	0.273	0.268	0.266	0.265	0.268
'M+3'	0.133	0.140	0.145	0.141	0.146	0.142	0.125	0.135	0.128	0.123	0.124	0.127
'M+4'	0.156	0.154	0.160	0.147	0.162	0.148	0.071	0.082	0.072	0.068	0.069	0.072
'M+5'	0.049	0.050	0.057	0.050	0.059	0.051	0.020	0.025	0.021	0.020	0.019	0.021
'M+6'	0.024	0.024	0.027	0.022	0.028	0.023	0.006	0.008	0.007	0.006	0.006	0.006
'M+7'	0.006	0.006	0.008	0.006	0.008	0.006	0.001	0.002	0.001	0.001	0.001	0.001
'M+8'	0.001	0.001	0.001	0.001	0.002	0.001	0.000	0.000	0.000	0.000	0.000	0.000
'M+9'	0.000	0.000	0.000	0.000	0.000	0.000	0.000	0.000	0.000	0.000	0.000	0.000
'Phenylalanine [M-159]'												
'M+0'	0.222	0.216	0.194	0.220	0.192	0.220	0.320	0.287	0.313	0.320	0.321	0.313
'M+1'	0.126	0.134	0.134	0.143	0.131	0.142	0.256	0.249	0.256	0.256	0.259	0.256
'M+2'	0.318	0.312	0.308	0.309	0.307	0.307	0.244	0.255	0.247	0.246	0.245	0.248
'M+3'	0.124	0.130	0.139	0.131	0.138	0.133	0.107	0.120	0.109	0.106	0.106	0.110
'M+4'	0.145	0.142	0.150	0.135	0.153	0.135	0.054	0.065	0.056	0.053	0.052	0.055
'M+5'	0.039	0.040	0.045	0.039	0.047	0.039	0.014	0.018	0.014	0.014	0.013	0.014
'M+6'	0.020	0.020	0.023	0.018	0.024	0.019	0.004	0.005	0.004	0.004	0.004	0.004
'M+7'	0.005	0.004	0.005	0.004	0.006	0.004	0.001	0.001	0.001	0.001	0.001	0.001
'M+8'	0.000	0.001	0.001	0.001	0.001	0.001	0.000	0.001	0.000	0.000	0.000	0.000
'Phenylalanine f302'												
'M+0'	0.604	0.595	0.572	0.605	0.564	0.605	0.699	0.665	0.692	0.707	0.703	0.691
'M+1'	0.374	0.378	0.396	0.369	0.400	0.369	0.282	0.309	0.288	0.277	0.278	0.289
'M+2'	0.022	0.026	0.032	0.026	0.036	0.026	0.019	0.026	0.020	0.017	0.019	0.020
'Aspartate [M-57]'												
'M+0'	0.366	0.349	0.334	0.337	0.346	0.333	0.371	0.361	0.369	0.375	0.373	0.371
'M+1'	0.194	0.211	0.221	0.227	0.225	0.229	0.320	0.319	0.320	0.318	0.323	0.322
'M+2'	0.306	0.298	0.296	0.288	0.288	0.286	0.214	0.221	0.217	0.215	0.214	0.215
'M+3'	0.100	0.108	0.114	0.114	0.110	0.117	0.080	0.084	0.081	0.079	0.077	0.079
'M+4'	0.034	0.034	0.036	0.034	0.032	0.035	0.014	0.014	0.013	0.013	0.013	0.012

'Aspartate [M-159]'												
'M+0'	0.423	0.410	0.394	0.403	0.420	0.402	0.453	0.443	0.452	0.459	0.458	0.453
'M+1'	0.213	0.230	0.241	0.248	0.238	0.246	0.325	0.325	0.326	0.323	0.325	0.326
'M+2'	0.289	0.281	0.281	0.270	0.269	0.271	0.180	0.187	0.181	0.178	0.177	0.181
'M+3'	0.074	0.078	0.084	0.079	0.073	0.081	0.041	0.044	0.041	0.040	0.039	0.041
'Aspartate f302'"												
'M+0'	0.526	0.519	0.508	0.517	0.534	0.516	0.588	0.579	0.585	0.594	0.592	0.588
'M+1'	0.337	0.336	0.339	0.332	0.329	0.335	0.304	0.308	0.306	0.302	0.304	0.306
'M+2'	0.136	0.145	0.153	0.151	0.137	0.149	0.108	0.113	0.109	0.104	0.104	0.106
'Glutamate [M-57]'												
'M+0'	0.239	0.232	0.223	0.222	0.220	0.216	0.288	0.286	0.293	0.287	0.298	0.296
'M+1'	0.167	0.179	0.186	0.190	0.188	0.190	0.297	0.298	0.300	0.298	0.304	0.304
'M+2'	0.319	0.311	0.307	0.305	0.306	0.305	0.251	0.252	0.248	0.252	0.246	0.246
'M+3'	0.142	0.149	0.154	0.155	0.156	0.160	0.117	0.116	0.114	0.115	0.110	0.111
'M+4'	0.108	0.103	0.105	0.101	0.104	0.102	0.040	0.041	0.039	0.041	0.036	0.037
'M+5'	0.025	0.025	0.026	0.027	0.027	0.027	0.007	0.007	0.007	0.008	0.006	0.006
'Glutamate [M-159]'												
'M+0'	0.284	0.278	0.266	0.268	0.265	0.263	0.350	0.353	0.359	0.351	0.368	0.362
'M+1'	0.192	0.204	0.211	0.216	0.214	0.218	0.326	0.326	0.328	0.325	0.330	0.329
'M+2'	0.331	0.324	0.323	0.320	0.322	0.320	0.224	0.223	0.220	0.226	0.214	0.217
'M+3'	0.103	0.109	0.114	0.114	0.116	0.117	0.079	0.078	0.075	0.078	0.070	0.073
'M+4'	0.090	0.085	0.086	0.082	0.084	0.082	0.021	0.020	0.019	0.021	0.017	0.018
'Histidine [M-57]'												
'M+0'	0.115	0.114	0.115	0.116	0.115	0.116	0.164	0.164	0.163	0.163	0.164	0.163
'M+1'	0.274	0.294	0.301	0.301	0.287	0.298	0.450	0.449	0.447	0.447	0.451	0.446
'M+2'	0.282	0.282	0.285	0.283	0.282	0.281	0.258	0.258	0.258	0.259	0.257	0.257
'M+3'	0.182	0.176	0.173	0.171	0.178	0.175	0.095	0.096	0.096	0.096	0.094	0.098
'M+4'	0.102	0.095	0.090	0.091	0.097	0.092	0.029	0.029	0.031	0.029	0.028	0.031
'M+5'	0.041	0.036	0.033	0.034	0.038	0.034	0.004	0.004	0.005	0.005	0.004	0.005
'M+6'	0.003	0.004	0.003	0.004	0.004	0.004	0.001	0.000	0.001	0.001	0.001	0.001
'Histidine [M-159]'												
'M+0'	0.128	0.126	0.128	0.128	0.130	0.128	0.179	0.182	0.180	0.181	0.181	0.180
'M+1'	0.330	0.349	0.354	0.354	0.345	0.350	0.483	0.487	0.481	0.484	0.484	0.478
'M+2'	0.318	0.319	0.321	0.317	0.316	0.317	0.260	0.258	0.258	0.260	0.257	0.258
'M+3'	0.165	0.149	0.145	0.145	0.152	0.147	0.053	0.052	0.057	0.054	0.054	0.057
'M+4'	0.049	0.043	0.041	0.042	0.046	0.044	0.013	0.012	0.013	0.013	0.013	0.015
'M+5'	0.010	0.013	0.011	0.013	0.012	0.014	0.011	0.008	0.011	0.008	0.009	0.012
