# Supplementary material for: Re-Programing Glucose Catabolism in the Microalga Chlorella sorokiniana under Light Condition
Source: Biomolecules. 2022 Jul 4;12(7):939. doi: 10.3390/biom12070939 (PMC9313030; doi:10.3390/biom12070939)
Supplement: Supplementary file 1 [file biomolecules-12-00939-s001.zip › Supplementary Materials, File S2.pdf]

Supplementary Materials, File S2. Metabolic reactions **in** ***C. sorokiniana***

	Reactions
1	'Glucose + ATP == G6P'
2	'G6P == F6P'
3	'F6P + ATP == FBP'
4	'FBP == DHAP + GAP'
5	'DHAP == GAP'
6	'GAP == 3PG + ATP + NADH'
7	'3PG == PEP'
8	'PEP == PYR + ATP'
9	'PYR == AceCoA + CO_2 + NADH'
10	'AceCoA + OAA == CIT'
11	'CIT == ICIT'
12	'ICIT == AKG + CO_2 + NADPH'
13	'AKG == SucCoA + CO_2 + NADH'
14	'SucCoA == SUC + ATP'
15	'SUC == FUM + FADH2'
16	'FUM == MAL'
17	'MAL == OAA + NADH'
18	'MAL == PYR + CO_2+ NADPH'
19	'PEP + CO_2 == OAA'
20	'G6P == Ru5P + CO2'
21	'Ru5P == RuBP'
22	'RuBP + CO2 == 2*3PG'
23	'X5P == Ru5P'
24	'R5P == Ru5P'
25	'GAP + S7P == X5P + R5P'
26	'E4P + F6P == GAP + S7P'
27	'E4P + DHAP == S7P'
28	'GAP + F6P == X5P + E4P'
29	'ICIT == GLX + SUC'
30	'GLX + AceCoA == MAL'
31	'RuBP + O2 == GLX + 3PG'
32	'GLX == GLY'
33	'3PG == SER'
34	'SER == GLY + C1'
35	'GLY == CO2 + C1'

36	'2*1.698*G6P+0.462*R5P+0.393*C1+0.347*GLY+0.199*SER+1.293*PYR+0.144 *E4P+0.638*GAP+0.779*PEP+7.682*AceCoA+0.494*AKG+0.261*OAA==Biomass'
