# Supplementary material for: Re-Programing Glucose Catabolism in the Microalga Chlorella sorokiniana under Light Condition
Source: Biomolecules. 2022 Jul 4;12(7):939. doi: 10.3390/biom12070939 (PMC9313030; doi:10.3390/biom12070939)
Supplement: Supplementary file 1 [file biomolecules-12-00939-s001.zip › Supplementary Materials, File S3.pdf]

Supplementary Materials, File S3. Relative flux distributions in *C. sorokiniana*.

Culture conditions	Mixotrophic conditions				Heterotrophic conditions			
Reactions	best fit	95LB	95UB	stev	best fit	95LB	95UB	stdv
'Glucose == G6P'	100	100	100	0	100	100	100	0
'G6P == F6P'	36	32	46	3	-11	-20	-2	5
'F6P == FBP'	39	34	128	24	89	71	105	9
'FBP == DHAP + GAP'	39	34	128	24	89	71	105	9
'DHAP == GAP'	25	16	40	6	44	35	47	3
GAP == 3PG'	37	15	73	15	112	90	117	7
3PG == PEP'	117	113	122	2	108	103	113	3
'PEP == PYR'	97	84	105	5	81	75	88	3
PYR == AceCoA + CO_2'	89	86	100	3	88	83	104	5
'OAA + AceCoA == CIT'	14	11	25	4	34	25	46	5
'CIT == ICIT'	14	11	25	4	34	25	46	5
ICIT == AKG + CO_2'	12	9	22	3	34	24	46	6
AKG == SucCoA + CO_2'	7	4	18	4	30	20	43	6
'SucCoA == SUC'	7	4	18	4	30	20	43	6
'SUC == FUM'	9	6	21	4	30	21	43	5
'FUM == MAL'	9	6	21	4	30	21	43	5
'MAL == OAA'	4	-4	12	4	14	7	25	5
MAL == PYR + CO_2'	5	0	24	6	16	10	38	7
PEP + CO_2 == OAA'	12	7	28	5	21	15	28	3
G6P == Ru5P + CO2'	31	23	35	3	88	77	97	5
Ru5P == RuBP'	42	23	55	8	0	0	17	4
RuBP + CO2 == 2*3PG'	42	23	55	8	0	0	0	0
X5P == Ru5P'	11	-3	21	6	-56	-62	-45	5
R5P == Ru5P'	0	-7	5	3	-32	-35	-26	2
GAP + S7P == X5P + R5P'	5	-2	10	3	-28	-32	-23	2
E4P + F6P == GAP + S7P'	-9	-100	-6	24	-73	-93	-54	10
E4P + DHAP == S7P'	14	12	108	25	44	27	62	9
GAP + F6P == X5P + E4P'	6	-1	11	3	-27	-31	-22	2
ICIT == GLX + SUC'	2	1	5	1	0	0	8	2
GLX + AceCoA == MAL'	0	0	8	2	0	0	21	5
RuBP + O2 == GLX + 3PG'	0	0	5	1	0	0	17	4
GLX == GLY'	2	1	3	1	0	0	0	0
3PG == SER'	5	4	5	0	4	4	4	0
SER == GLY + C1'	3	2	3	0	3	2	3	0
GLY == CO2 + C1'	1	0	2	0	0	0	0	0
2*1.698*G6P+0.462*R5P+0.393*C1+0.347*GLY+0.199*SER+1.293 *PYR+0.144*E4P+0.638*GAP+0.779*PEP+7.682*AceCoA+0.494 *AKG+0.261*OAA==Biomass'	10	9	10	0	7	6	8	0
Reversibility coefficient of reaction 'G6P == F6P'	1.00	0.76	1.00	0.06	0.40	0.32	0.69	0.09
Reversibility coefficient of reaction 'F6P == FBP'	0.66	0.01	0.70	0.18	0.05	0.01	0.47	0.12
Reversibility coefficient of reaction 'FBP == DHAP + GAP'	0.57	0.00	0.72	0.18	0.01	0.01	0.47	0.12
Reversibility coefficient of reaction 'DHAP == GAP'	0.49	0.37	0.99	0.16	0.67	0.08	0.93	0.22
Reversibility coefficient of reaction 'GAP == 3PG'	1.00	0.62	1.00	0.10	0.69	0.50	1.00	0.13
Reversibility coefficient of reaction '3PG == PEP'	1.00	0.29	1.00	0.18	0.53	0.00	1.00	0.25

Reversibility coefficient of reaction 'PEP == PYR'	0.12	0.00	1.00	0.25	0.00	0.00	0.52	0.13
Reversibility coefficient of reaction 'CIT == ICIT'	0.51	0.46	0.54	0.02	0.50	0.48	0.53	0.01
Reversibility coefficient of reaction 'ICIT == AKG + CO2'	0.00	0.00	0.93	0.24	0.48	0.04	0.54	0.13
Reversibility coefficient of reaction 'SucCoA == SUC'	0.50	0.44	0.57	0.03	0.50	0.43	0.56	0.03
Reversibility coefficient of reaction 'SUC == FUM'	0.49	0.44	0.59	0.04	0.54	0.43	0.56	0.03
Reversibility coefficient of reaction 'FUM == MAL'	0.83	0.44	1.00	0.14	0.92	0.32	1.00	0.17
Reversibility coefficient of reaction 'MAL == OAA'	0.84	0.49	1.00	0.13	0.92	0.74	1.00	0.07
Reversibility coefficient of reaction 'PEP + CO2 == OAA'	0.04	0.00	0.57	0.14	0.00	0.00	0.76	0.19
Reversibility coefficient of reaction 'X5P == Ru5P'	0.25	0.00	0.99	0.25	0.51	0.28	0.80	0.13
Reversibility coefficient of reaction 'R5P == Ru5P'	1.00	0.58	1.00	0.11	0.83	0.20	0.99	0.20
Reversibility coefficient of reaction 'GAP + S7P == X5P + R5P'	0.99	0.02	1.00	0.25	0.51	0.20	1.00	0.20
Reversibility coefficient of reaction 'E4P + F6P == GAP + S7P'	0.00	0.00	0.61	0.16	0.20	0.00	0.72	0.18
Reversibility coefficient of reaction 'GAP + F6P == X5P + E4P'	0.09	0.00	0.40	0.10	0.00	0.00	0.05	0.01
Reversibility coefficient of reaction 'SER == GLY + C1'	0.00	0.00	0.31	0.08	0.00	0.00	0.01	0.00
Reversibility coefficient of reaction 'GLY == CO2 + C1'	0.00	0.00	0.01	0.00	0.00	0.00	0.00	0.00

Note: (1) All values are normalized to the glucose uptake rate, which was assumed to be 100;

(2) 95 LB and UB indicate 95% confidence interval lower and upper bound.
